# Supplementary material for: Expanding the CRISPR Toolbox with ErCas12a in Zebrafish and Human Cells
Source: CRISPR J. 2019 Dec 16;2(6):417–33. doi: 10.1089/crispr.2019.0026 (PMC6919245; doi:10.1089/crispr.2019.0026)
Supplement: Supplemental data [file Supp_Fig1.pdf]

Supplementary Data

SUPPLEMENTARY FIG. S1. Amino acid alignment of Cas12a family members with SpCas9. Multiple sequence alignment of ErCa12a with homologous Cas proteins, including CRISPR-associated endonuclease Cas12b from *Alicyclobacillus acidoterrestris*, CRISPR-associated Endonuclease Cas9 from *Streptococcus pyogens*, CRISPR-associated Endonuclease Cpf1 from *Acidaminococcus* sp. BV3L6, type V CRISPR-associated protein Cpf1 from *Lachnospiraceae* bacterium ND2006, and type V CRISPR-associated protein Cpf1 from *Francisella tularensis.* Six representative sequences were used for alignments with Clustal Omega webserver.

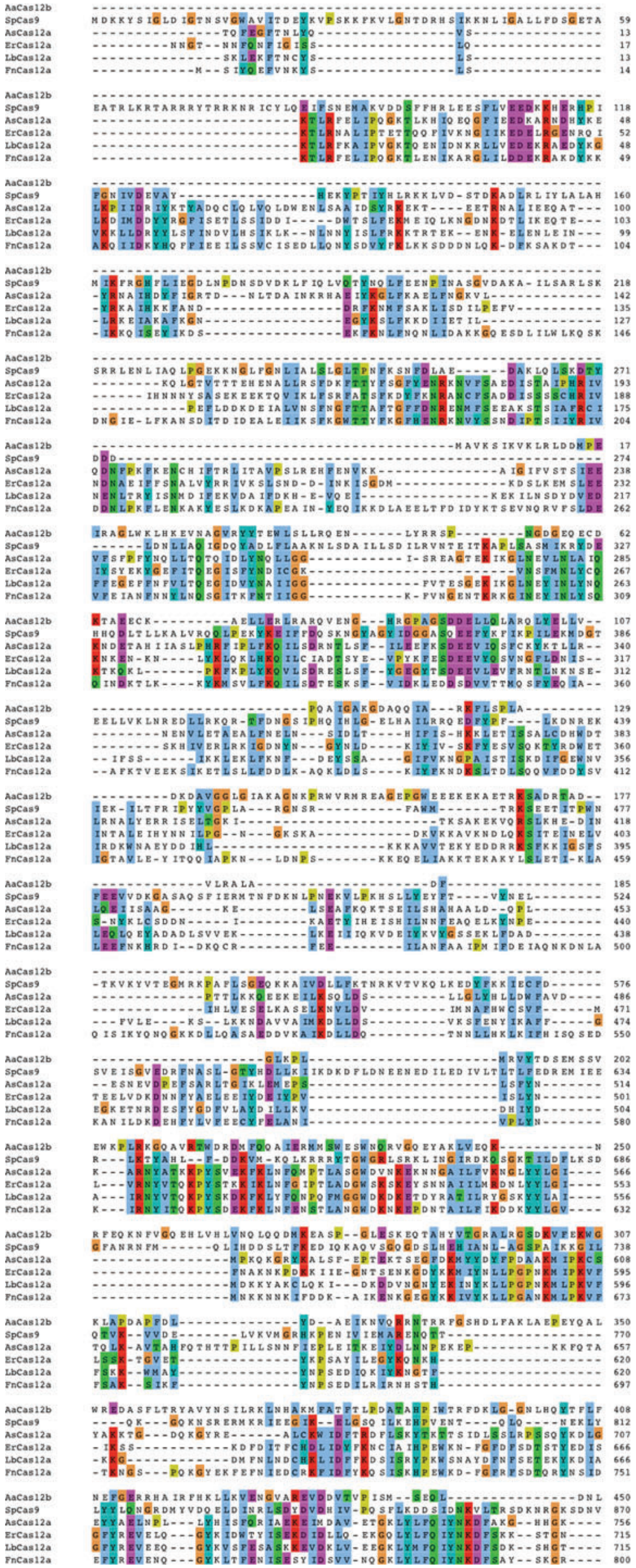
AaCas12b
SpCas9
AsCas12a
ErCas12a
LbCas12a
FnCas12a

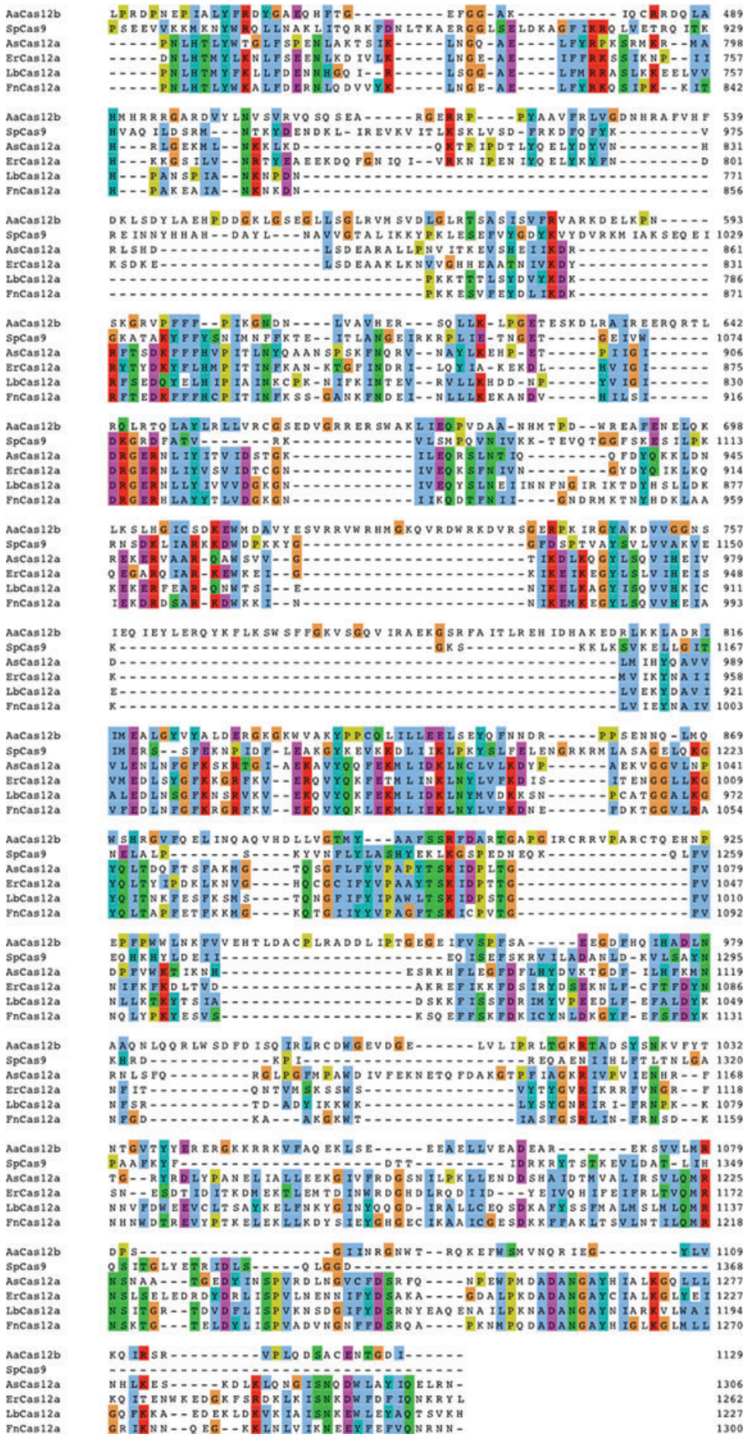

SUPPLEMENTARY FIG. S1. (Continued).
