# Supplementary material for: Expanding the CRISPR Toolbox with ErCas12a in Zebrafish and Human Cells
Source: CRISPR J. 2019 Dec 16;2(6):417–33. doi: 10.1089/crispr.2019.0026 (PMC6919245; doi:10.1089/crispr.2019.0026)
Supplement: Supplemental data [file Supp_Fig2.pdf]

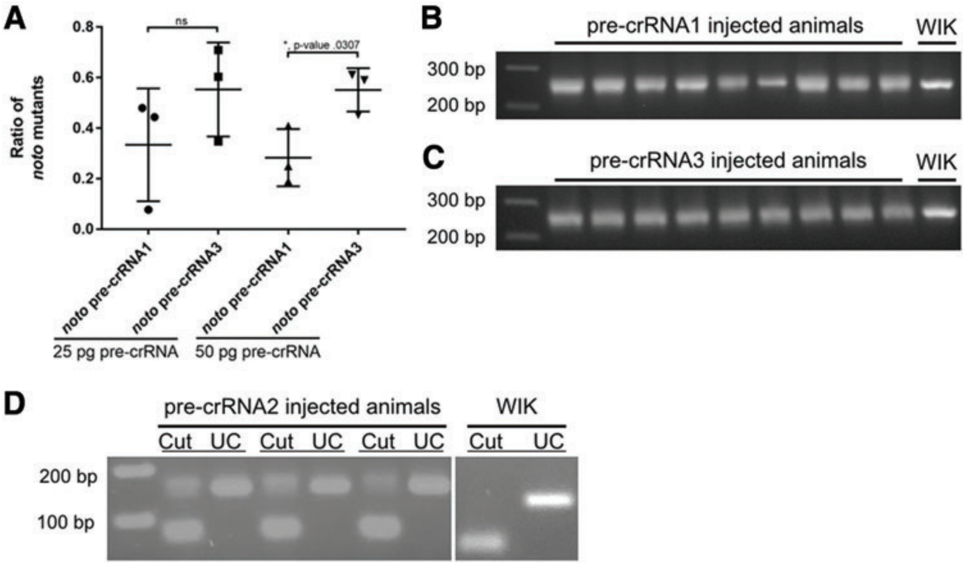

SUPPLEMENTARY FIG. S2. Qualitative representation of ErCas12a activity in zebrafish embryos. **(A)** Data plots showing ratio of injected animals displaying somatic *noto* phenotype. Data plot represents the mean ± *SD*. *p*-Values calculated with one-tailed Student's *t*-test. **(B** and **C)** Gel showing heteroduplex mobility shift of injected animals versus wild type (WIK) for pre-crRNA1 and pre-crRNA3 at *noto*. **(D)** RFLP analysis showing pre-crRNA2 is active at *cx43.4*.
