# Supplementary material for: Expanding the CRISPR Toolbox with ErCas12a in Zebrafish and Human Cells
Source: CRISPR J. 2019 Dec 16;2(6):417–33. doi: 10.1089/crispr.2019.0026 (PMC6919245; doi:10.1089/crispr.2019.0026)
Supplement: Supplemental data [file Supp_Fig3.pdf]

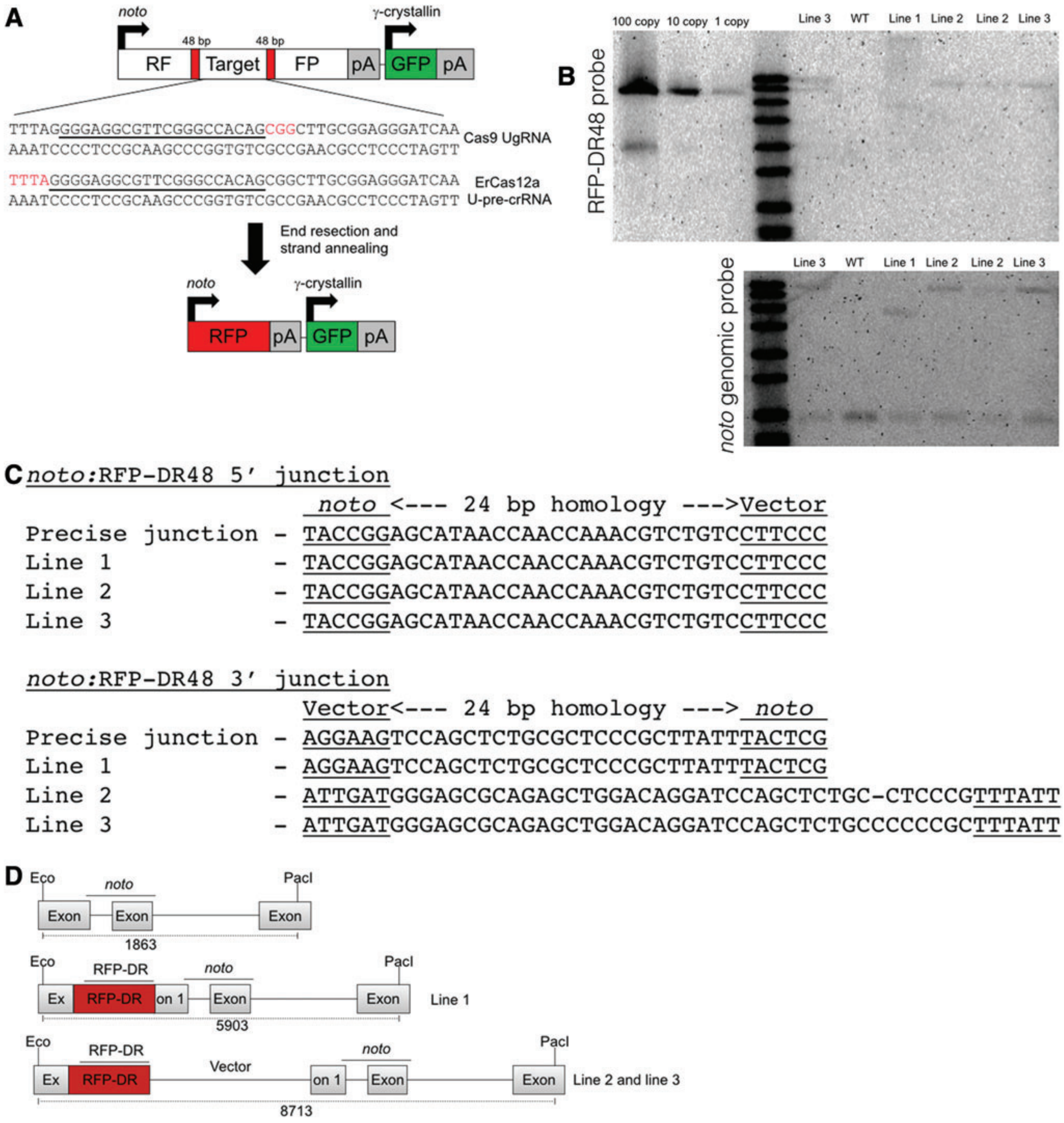

SUPPLEMENTARY FIG. S3. Engineering *noto*:RFP-DR48. **(A)** Schematic showing RFP-DR48 integrated at *noto*. The NBM (yellow) contains both SpCas9 and ErCas12a PAMs for universal targeting using the underlined RNA cursor target site. Red is the engineered stop codon. **(B)** Southern blot of three *noto*:RFP-DR48 lines. Band intensity is roughly single copy based on the copy number controls in the *noto* probed blot (top). Gel shifts are present indicating precise (line 1) and linear vector integration (lines 2 and 3). **(C)** Junction fragment analysis of integrations showing precision at the 5′ junction. Line 1 contains a precise 3′ integration, while line 2 and line 3 contain differing NHEJ events. **(D)** Schematic of integrations events as determined by Southern blot and DNA sequencing analysis.
