# Supplementary material for: Expanding the CRISPR Toolbox with ErCas12a in Zebrafish and Human Cells
Source: CRISPR J. 2019 Dec 16;2(6):417–33. doi: 10.1089/crispr.2019.0026 (PMC6919245; doi:10.1089/crispr.2019.0026)
Supplement: Supplemental data [file Supp_Fig4.pdf]

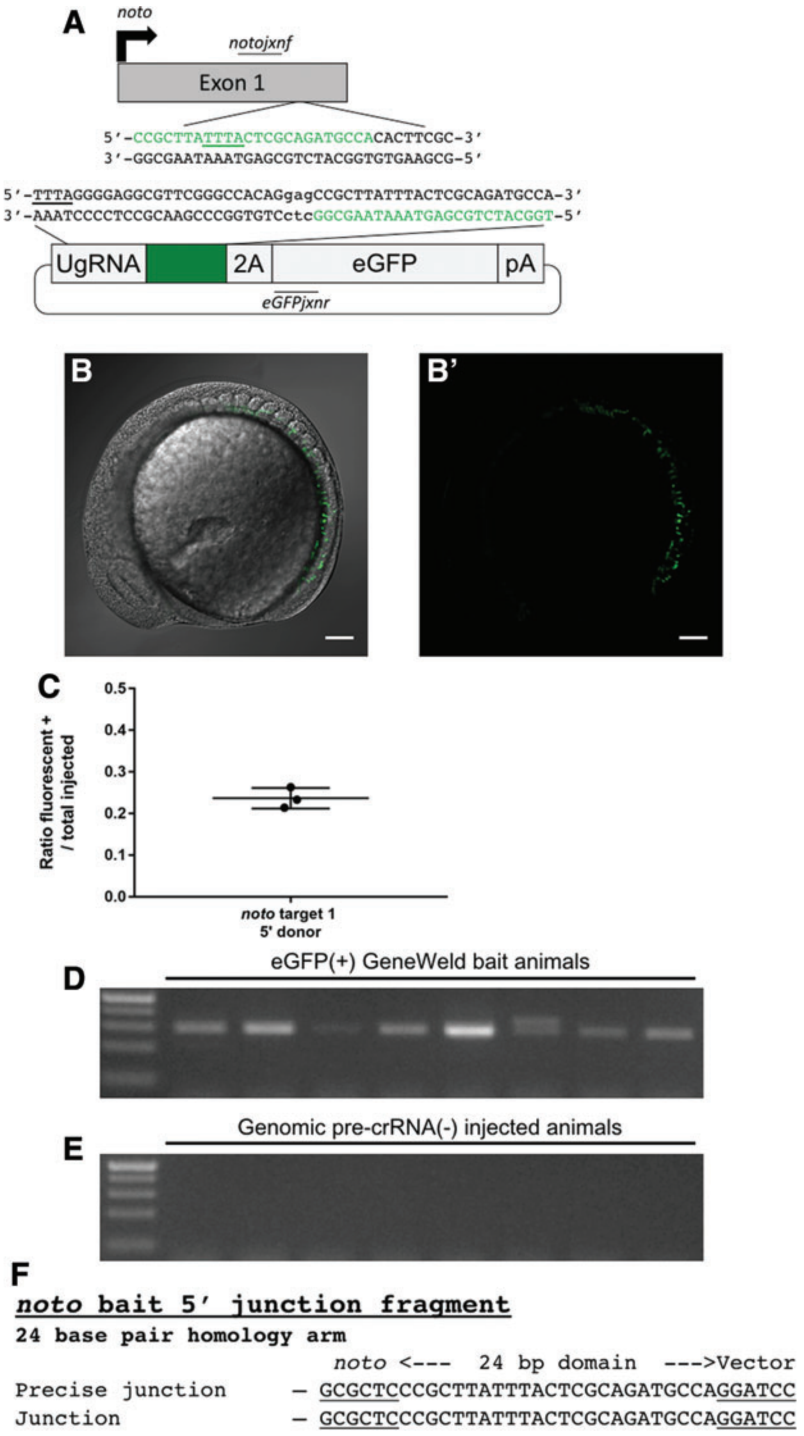

SUPPLEMENTARY FIG. S4. Targeting *noto* with a single homology domain. **(A)** Schematic showing designed homology for precise 5′ integration using ErCas12a. Green is designed homology. The PAM for ErCas12a targeting in the genome and donor is underlined. **(B** and **B′)** Representative confocal Z-stack image showing mosaic GFP expression in the notochord of an injected animal. Scale bar: 100 μm. **(C)** Data plot showing the ratio of embryos with GFP expression in the notochord out of total injected embryos. Data plot represents the mean $\pm$ *SD*. **(D)** Gel showing junction fragment expected after precise integration using the homology domain. **(E)** Gel showing no junction, indicating there is no integration without the genomic pre-crRNA. **(F)** DNA sequencing 5′ junctions showing precise integration using the programmed homology.
