# Supplementary material for: Expanding the CRISPR Toolbox with ErCas12a in Zebrafish and Human Cells
Source: CRISPR J. 2019 Dec 16;2(6):417–33. doi: 10.1089/crispr.2019.0026 (PMC6919245; doi:10.1089/crispr.2019.0026)
Supplement: Supplemental data [file Supp_Fig5.pdf]

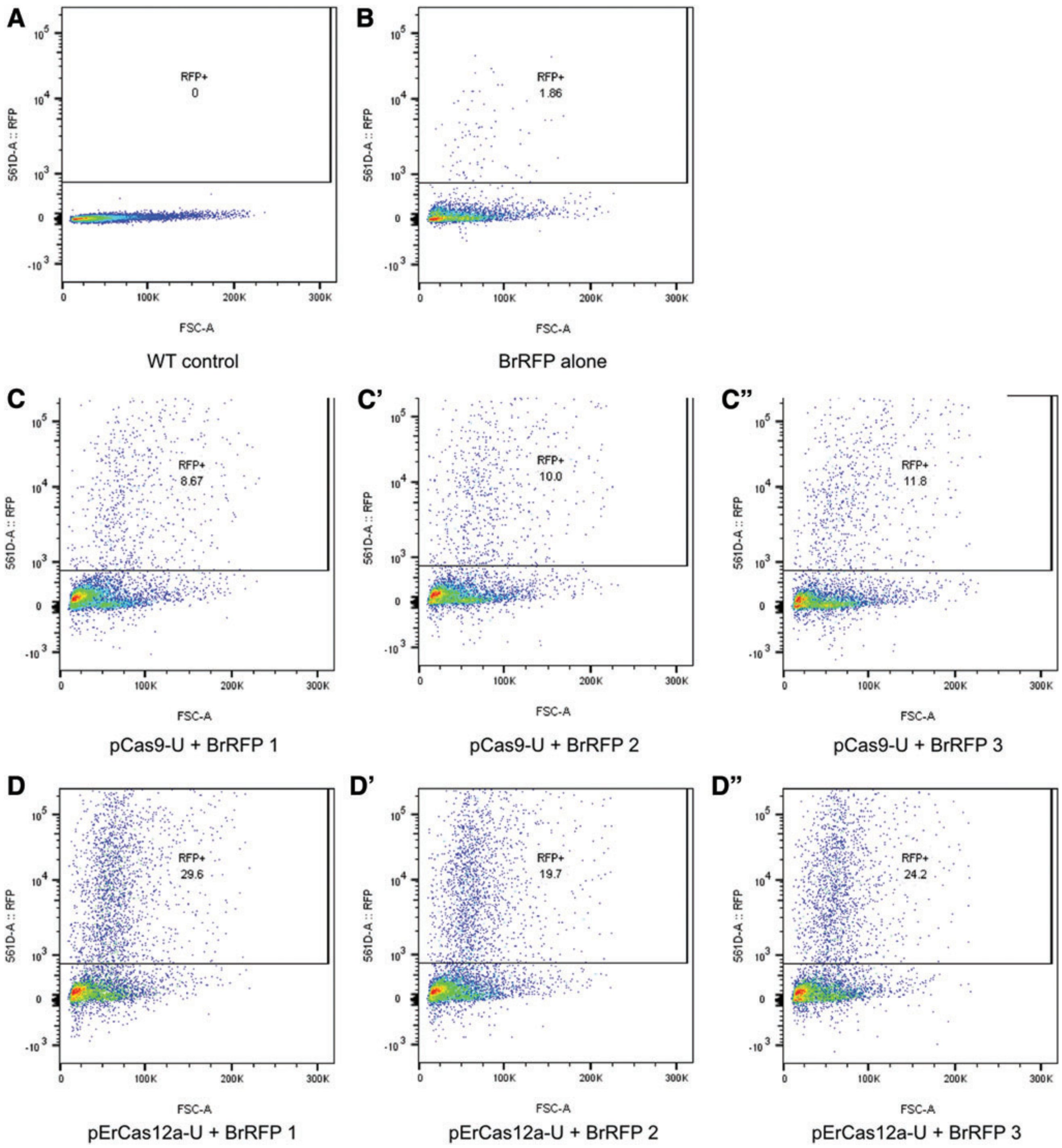

SUPPLEMENTARY FIG. S5. Raw data for flow cytometry of RFP-DR48 in HEK293T cells. **(A)** Untransfected HEK293T cells. **(B)** HEK293T cells transfected with pMini-CAAGs::RFP-DR48. **(C-C")** HEK293T cells transfected with SpCas9-UgRNA and pMini-CAAGs::RFP-DR48. **(D-D")** HEK293T cells transfected with ErCas12a-pre-U-crRNA. The RFP plots shown were gated on the single-cell population. All measurements taken using excitation of 584 nm and emission of 607 nm.
