# Supplementary material for: Expanding the CRISPR Toolbox with ErCas12a in Zebrafish and Human Cells
Source: CRISPR J. 2019 Dec 16;2(6):417–33. doi: 10.1089/crispr.2019.0026 (PMC6919245; doi:10.1089/crispr.2019.0026)
Supplement: Supplemental data [file Supp_Fig6.pdf]

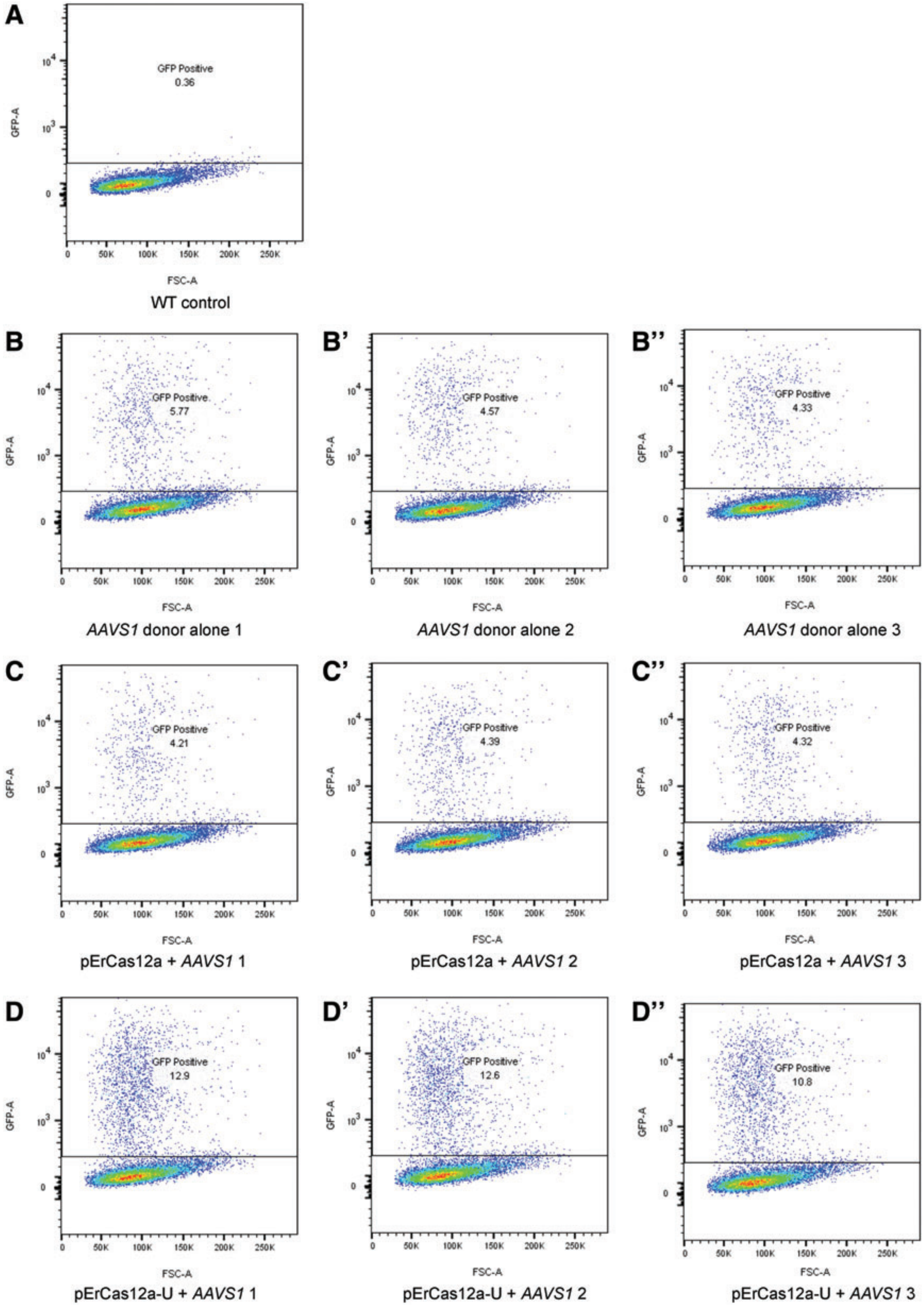

SUPPLEMENTARY FIG. S6. Raw data for flow cytometry of GeneWeld in HEK293T cells. **(A)** Untransfected HEK293T cells. **(B–B″)** HEK293T cells transfected only with the GFP GeneWeld donor. **(C–C″)** HEK293T cells transfected with the GFP GeneWeld donor and pErCas12a-AAVS1. **(D–D″)** HEK293T cells transfected with the GFP GeneWeld donor and P-ErCas12a-U-AAVS1. The GFP plots shown were gated on the single-cell population. All measurements taken using the FITC channel and excitation of 488 nm and emission of 510 nm.
